# Qualitative Study on the Observations of Emissions, Transport, and the Influence of Climatic Factors from Sugarcane Burning: A South African Perspective

**DOI:** 10.3390/ijerph18147672

**Published:** 2021-07-19

**Authors:** Lerato Shikwambana, Xolile Ncipha, Sivakumar Kandasami Sangeetha, Venkataraman Sivakumar, Paidamwoyo Mhangara

**Affiliations:** 1 Earth Observation Directorate, South African National Space Agency, Pretoria 0001, South Africa; 2 School of Geography, Archaeology and Environmental Studies, University of the Witwatersrand, Johannesburg 2050, South Africa; paida.mhangara@wits.ac.za; 3 South African Weather Service, Private Bag X097, Pretoria 0001, South Africa; xolile.ncipha@weathersa.co.za; 4 School of Chemistry and Physics, University of KwaZulu-Natal, Durban 4041, South Africa; trijashan@yahoo.com (S.K.S.); Venkataramans@ukzn.ac.za (V.S.)

**Keywords:** smoke, black carbon, HYSPLIT model, biomass burning, meteorology, sequential Mann–Kendall

## Abstract

There are two methods of harvesting sugarcane—manual or mechanical. Manual harvesting requires the burning of the standing sugarcane crop. Burning of the crop results in the emission of aerosols and harmful trace gases into the atmosphere. This work makes use of a long-term dataset (1980–2019) to study (1) the atmospheric spatial and vertical distribution of pollutants; (2) the spatial distribution and temporal change of biomass emissions; and (3) the impact/influence of climatic factors on temporal change in atmospheric pollutant loading and biomass emissions over the Mpumalanga and KwaZulu Natal provinces in South Africa, where sugarcane farming is rife. Black carbon (BC) and sulfur dioxide (SO_2_) are two dominant pollutants in the JJA and SON seasons due to sugarcane burning. Overall, there was an increasing trend in the emissions of BC, SO_2_, and carbon dioxide (CO_2_) from 1980 to 2019. Climatic conditions, such as warm temperature, high wind speed, dry conditions in the JJA, and SON season, favor the intensity and spread of the fire, which is controlled. The emitted pollutants are transported to neighboring countries and can travel over the Atlantic Ocean, as far as ~6600 km from the source site.

## 1. Introduction

Sugarcane (*Saccharum officinarum*) is a crop that produces sugar and is primarily grown in tropical countries. It has the distinguished quality of serving as both a food and a fuel source. Sugar is said to be an important source of energy for the body; however, the World Health Organization (WHO) recommends that adults and children reduce their daily intake of sugar to less than 10% of their total energy intake. Studies have shown that consuming excess sugar can increase unhealthy weight gain, heighten risk of diseases (such as diabetes, high blood pressure, heart disease), and can damage teeth, causing dental caries. Having stated these facts, it must be noted that the sugar industry is one of the world’s oldest, with a strong influence on the economic, political, and societal developments around the world [1,2]. Worldwide, the production of sugar between 2009 and 2019 grew by 12.79 million metric tons [3]. This illustrates that the demand for sugar is on the rise with a growing global population.

In 2013, global production of sugarcane was estimated to be 1.9 Gt, with Brazil accounting for 39%, India accounting for 18%, and sub-Saharan Africa (SSA) accounting for 4% of the global production [4]. South Africa (23%), Sudan (including South Sudan) (9%), Kenya (7%), Swaziland (7%), and Mauritius (7%) are the five countries that account for more than half of the total production of sugarcane in SSA [4]. South Africa is the most producing sugarcane country in SSA, with 14 cane-producing areas, extending from Northern Pondoland in the Eastern Cape Province through the coastal belt and Kwa- Zulu-Natal midlands to the Mpumalanga Lowveld. The sugar industry in South Africa burns about 90% of its crop at harvest while 10% is harvested green [5]. There are some disadvantages and advantages of burning sugarcane. Some of the disadvantages of burning is that (1) ashes from the fire fall in areas, such as schools, residential areas, and beaches; (2) smoke from the fires cause hazards to road users and the general public; and (3) burnings under high tension power lines sometimes cause a short, disrupting the power supply [5]. Some of the advantages of sugarcane burning are that (1) burning reduces the weight of the harvested crop, which means transport costs are lower; and (2) the flames drive away cane rats and snakes that can pose a threat to workers [6].

Burning of sugarcane and stalks is another type of biomass burning (BB) [7,8]. BB emits large amounts of carbon monoxide (CO), CO_2_, smoke, particulate matter (PM), and other constituents into the atmosphere. BB is the largest source of primary fine carbonaceous particles and the second largest source of trace gases in the global atmosphere [9,10,11]. Trace gases emitted by BB have a significant influence on the atmosphere, which includes a major contribution to the formation of the global tropospheric ozone, which is a vital greenhouse gas [12,13]. Moreover, BB emissions have a negative impact on human health [14,15,16], the climate [16,17,18], and the environment [19]. Thus, having the appropriate tools (instruments) to measure these emissions will help mitigate these impacts better.

Open field burning of sugar cane for harvesting is widespread in South Africa and is considered as one of the main sources of greenhouse gases [20]. Pryor et al. [21] observed that pre-harvest burning of sugar cane fields in South Africa results in net greenhouse gas due to partial combustion and emission of nitrous oxide (N_2_O) and methane (CH_4_). Eustice et al. [22] highlights the paucity of research in the emissions, transport, and complex interaction of factors from sugarcane burning in South Africa and recommends that more detailed research is needed to understand measure emissions and their interactions from sugarcane burning in South Africa. Knowledge gaps still exist in understanding the complex interactions of gas emissions and transport dynamics from sugarcane burning. The impact of carbon dioxide (CO_2_), N_2_O, and CH_4_ is of primary concern, and researchers also underscore the need to understand the influence of precursor gases, such as CO, NOx, ammonia (NH_3_), and sulfur dioxide (SO_2_).

According to the South African Sugar Association (SASA) there has been an increase in the yields per hectare of harvested cane (in tons) from the 2005/2006 season (6602) to the 2018/2019 season (74,944) [23]. This large increase in yield implies larger BB emissions in the season of 2018/2019. Therefore, the main aim of this study is to investigate the long-term trends of constituents emitted from BB. The other objectives of the study are to (1) study the spatial and vertical distributions of pollutants; (2) study the dispersions of pollutants due to winds and air masses; and (3) study the impact/influence of climatic factors.

## 2. Study Area

**Figure 1:**
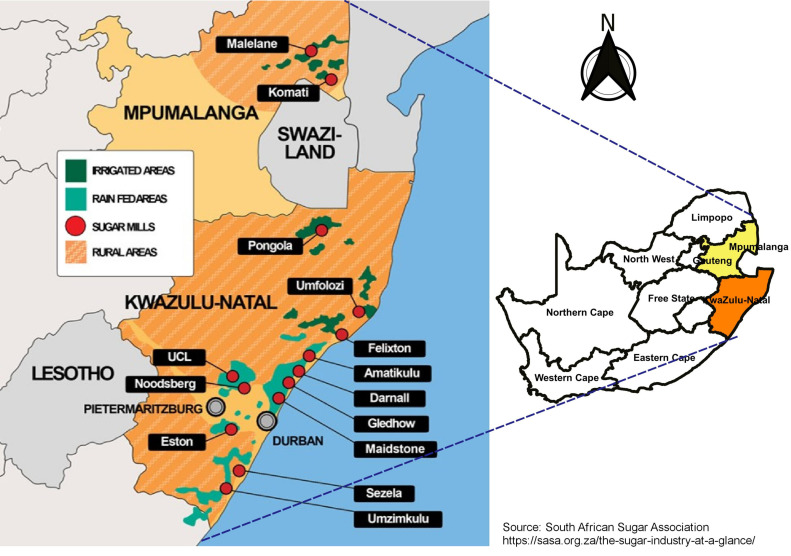
Sugarcane plantations regions in the Mpumalanga and KwaZulu-Natal provinces of South Africa.

Figure 1 shows a map of areas where sugarcane plantations and sugar mills are located in South Africa. There are currently 14 sugarcane milling companies in South Africa. Twelve of the mills are located in the KwaZulu-Natal (KZN) province and the other two in the Mpumalanga province (MP). KZN has the largest supply area with mostly rain-fed sugarcane (75%), while 25% of the sugarcane plantations in KZN are produced under irrigation. However, in MP, both sugarcane plantations are under irrigation. It must be noted that sugarcane in rain-fed areas is dependent on the specific climate conditions. Therefore, low rainfall or droughts have a negative impact on sugarcane production in those areas.

## 3. Data and Methods

In this study, various data sources and products, including satellite, reanalysis, and model data are used. Some advantages of using satellite data in this study are (1) the large area coverage, which enables regional surveys on a variety of themes and identification of extremely large features; (2) the easy collection of data over a variety of scales and resolutions; and (3) the repetitive coverage, which becomes convenient when collecting data on dynamic themes, such as BB emissions. Reanalysis datasets, on the other hand, are produced via data assimilation, a process that relies on both observations and model-based forecasts to estimate conditions [24]. Although there are some errors and uncertainties associated with reanalysis datasets, the datasets do provide crucial information on understanding systemic dynamics. In the following sub-sections, the datasets used in this study are briefly discussed.

### 3.1. MERRA-2

The Modern-Era Retrospective analysis for Research and Applications, Version 2 (MERRA-2) is an atmospheric reanalysis designed to provide an intermediate dataset and bridge between the first MERRA reanalysis [25] and the project’s long-term objective of producing a coupled Earth system reanalysis [26]. The grander feature of MERRA-2 over its predecessor, MERRA, is that it assimilates several observation types, and includes updates to the Goddard Earth Observing System (GEOS) model and analysis scheme; this allows it to provide a feasible ongoing climate analysis [27,28]. MERRA-2 incorporates system changes and fundamental developments in modeling and data assimilation, including (1) assimilation of aerosol observations that can interact with atmospheric radiative processes; (2) constraining mass conservation, even with the analysis of water vapor, allowing a global balance between evaporation and precipitation; (3) use of a cube sphere to reduce the effect of grid point singularities at the pole, allowing for improved polar circulation; (4) an updated radiative transfer model to permit the assimilation of data from many more instruments than could have been included in MERRA; and (5) inclusion of new observational forcing for the land model to provide more stable land feedback processes [26,29,30]. The model includes the finite-volume dynamical core of Putman and Lin [31], which uses a cubed sphere horizontal discretization at an approximate resolution of 0.5° × 0.625° and 72 hybrid-eta levels from the surface to 0.01 hPa [29]. The analysis is computed on a latitude–longitude grid at the same spatial resolution as the atmospheric model using a three-dimensional variational (3DVAR) algorithm based on the GSI with a 6-h update cycle and the so-called first-guess-at-appropriate-time (FGAT) procedure for computing temporally accurate observation-minus-background departures. The analysis is applied as a correction to the background state using an incremental analysis update (IAU) procedure [32]. A detailed description of the MERRA-2 aerosol analysis system and its validation are presented in Randles et al. [29] and Buchard et al. [30]. In this study, black carbon (BC) biomass burning emissions, organic carbon (OC) biomass burning emissions, sulfur dioxide (SO_2_) biomass burning emissions, BC aerosol optical depth (AOD), OC AOD, wind speed, and wind vectors are the parameters used.

### 3.2. CALIPSO

The Cloud-Aerosol Lidar and Infrared Pathfinder Satellite Observations (CALIPSO) was launched together with the CloudSat satellite in April 2006 [33]. CALIPSO was launched with the goal of filling existing gaps in our ability to observe the global distribution and properties of aerosols and clouds [34]. The orbit inclination of 98.2° provides global coverage from CALIPSO between 82° N and 82° S. The CALIPSO orbit is controlled to keep cross-track errors with respect to the 16-day grid less than ±10 km [35]. The primary instrument onboard the CALIPSO satellite are the Cloud-Aerosol Lidar with Orthogonal Polarization (CALIOP), the wide field camera (WFC) and the infrared imaging radiometer (IIR). CALIOP is an elastically backscattered LIDAR operating at 532 and 1064 nm, equipped with a depolarization channel at 532 nm. It can be categorized into two product levels: level 1 and level 2. The level 1 products are made up of calibrated and geolocated profiles of the attenuated backscatter returned signal. Level 2 products, in comparison, are derived from level 1 products and are classified in three types: profile, vertical feature mask, and layer products [36]. A detailed technical discussion of the CALIPSO is found in Winker et al. [34], and Winker et al. [37]. In this study, we use the elevated smoke extinction coefficient data.

### 3.3. AIRS

The Atmospheric Infrared Sounder (AIRS) is a hyperspectral instrument onboard the National Aeronautics and Space Administration’s (NASA) Earth Observing System (EOS) Aqua satellite. AIRS was launched in 2002 with the purpose of for climate studies on greenhouse gases and carbon dioxide distribution, as well as weather forecasts. The instrument is designed to collect climate data and turn it into 3D maps of air and surface temperature, water vapor, and cloud properties. It has the capacity to measure the atmospheric temperature in the troposphere with an accuracy of 1 K over 1 km-thick layers under both clear and cloudy conditions. AIRS has a cross-track scanning spectrometer with 2378 IR channels between 3.74 and 4.61 µm, 6.20–8.22 µm, and 8.8–15.5 μm, which is essential for atmospheric temperature and relative humidity soundings. AIRS also has four visible (VIS) and near-IR (NIR) channels between 0.40 and 0.94 μm, which are mainly used for the detection of clouds in the IR FOV. The IR resolution obtainable from AIRS is 13.5 km in the horizontal and 1 km in the vertical, and the VIS/NIR spatial resolution is ∼2.3 km. More details of the instrument are discussed by Aumann and Miller [38], Chahine et al. [39], and Menzel et al. [40]. In this study, we used the surface temperature and relative humidity data obtained from AIRS.

### 3.4. TRMM

The Tropical Rainfall Measuring Mission (TRMM) satellite was launched in November 1997. TRMM has five instruments onboard namely, the Precipitation Radar (PR), TRMM Microwave Imager (TMI), Visible and Infrared Scanner (VIRS), Clouds and the Earth’s Radiant Energy System (CERES), and the Lightning Imaging Sensor (LIS). The CERES instrument failed after only a few months of operation, but the other four instruments continue to operate, providing detailed information of rainfall over the tropics. The TMI and PR are the main instruments used for precipitation. The specifications of the TMI, PR, VIRS and LIS instruments can be found in Kummerow et al. [41] and Liu et al. [42]. Some of the highlights of TRMM are (1) its ability to give high spatial and temporal resolution precipitation estimates over a relatively long period of record since 1998; and (2) its expediency in investigating the climatological distribution of rainfall, and its frequency and intensity. In this study we used the precipitation rate datasets.

### 3.5. HYSPLIT Model

The Hybrid Single-Particle Lagrangian Integrated Trajectory model (HYSPLIT) model was developed by the National Oceanic and Atmospheric Administration (NOAA) Air Resources Laboratory and the Australian Bureau of Meteorology Research Center in 1998 [43]. The HYSPLIT model is a complete system for computing simple air parcel trajectories as well as complex transport, dispersion, chemical transformation, and deposition simulations [43]. One of the most common model applications is a back-trajectory analysis to determine the origin of air masses and establish source–receptor relationships [44]. The forward trajectory plots, on the other hand, are valuable for determining the receiving environment or impact area of a particular source [45]. The developmental history and technical aspects of the HYSPLIT model is found in Stein et al. [46]. One of the advantages of the HYSPLIT model is its ability to simulate scenarios describing the atmospheric transport, dispersion, and deposition of pollutants and hazardous materials. Some examples include the transport of volcanic aerosols [47] and wildfire smoke [48]. In this study, we used the back-trajectory analysis to establish the source-receptor relationship.

### 3.6. SQ–MK Test

The Sequential Mann–Kendall (SQ–MK) test proposed by Sneyers [49] was used to identify abrupt changes in significant trends [50,51]. This test sets up two series; a progressive $u\left(t\right)$ and a retrograde (backward) series $u\prime \left(t\right)$. If they cross each other and diverge beyond the specific threshold value, then there is a statistically significant trend. The point where they cross each other indicates the approximate year at which the trend begins [52]. The threshold values in this study are ±1.96 (*p* = 0.05), with the crossing point estimating the year at which the trend begins. The SQ–MK test has the following steps:

- At each comparison, the number of cases *x_i_* > *x_j_* is counted and indicated by $n_{i}$, where *x_i_* (*i* = 1, 2, … *n*) and *x_j_* (1, 2, … *n*) are the sequential values in a series, respectively.
- The test statistic *t_i_* is calculated by
  (1) $$t_{i}=\sum_{j=1}^{i}n_{j}$$
- The mean *E*(*t*) and the variance var(*t_i_*) of the test statistic are calculated by
  (2) $$E(t)=\frac{n(n-1)}{4}$$
  (3) $$\mathrm{var}(t_{i})=\frac{i(i-1)\left(2i+5\right)}{72}$$
- Sequential progressive value can be calculated as
  (4) $$u(t)=\frac{t_{i}-E(t)}{\sqrt{\mathrm{var}(t_{i})}}$$

Similarly, the values of $u\prime \left(t\right)$ are computed backward, starting from the end of series. In this study, SQ–MK was performed on BC biomass burning emissions, SO_2_ biomass burning emissions, OC biomass burning emissions, and CO_2_ concentration.

### 3.7. Surface Rainfall Data

The rainfall data presented in this study were collected from four automatic rainfall weather stations in the study region, bounded by the (29° to 30°) E longitude and (29° to 31°) S latitude. The stations are operated by the South African Weather Service (SAWS) national climate monitoring network. The sites were selected to show the spatial variations of the rainfall over the study area during the study period. In this study, the rainfall data were provided as an annual accumulation.

## 4. Results and Discussions

### 4.1. Emissions from Sugarcane Burning

**Figure 2:**
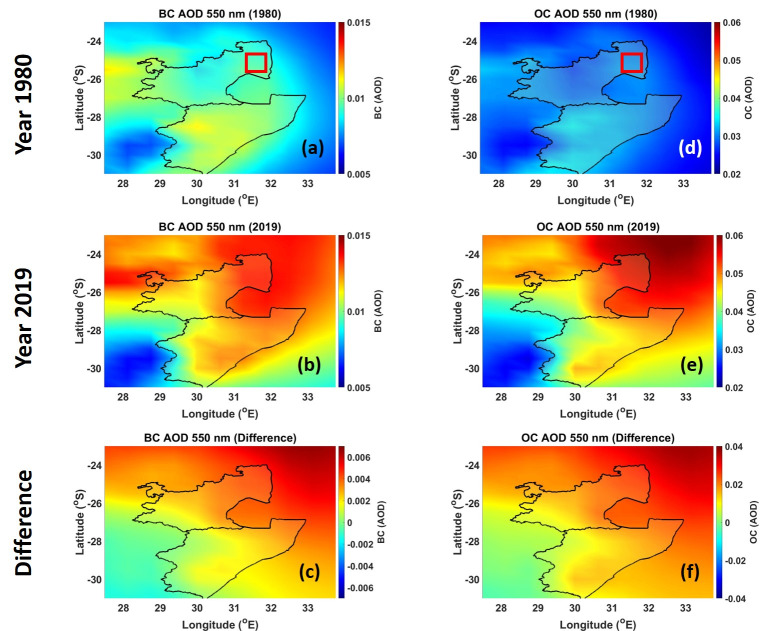
BC AOD over the KwaZulu Natal and Mpumalanga provinces during (**a**) 1980, (**b**) 2019, and (**c**) the difference between 2019 and 1980. OC AOD over the KwaZulu Natal and Mpumalanga provinces during (**d**) 1980, (**e**) 2019, and (**f**) the difference between 2019 and 1980.

The spatial distribution of BC AOD and OC AOD are shown in Figure 2. Based on the locations of the sugarcane plantations in Figure 1 and the distribution of the BC AOD and OC AOD in Figure 2, it can be concluded that most of the emissions observed arise from the sugarcane burning. The highest BC AOD in the year 1980 (see Figure 2a) was 0.02, which was observed in the KZN region and over the sugarcane plantations in the coast. The BC AOD on the coast can be attributed to sugarcane burning. In the MP region, where sugarcane plantations (red square) are supposed to be, the BC AOD values are low < 0.01 (see Figure 2a). This is because in the MP region sugarcane production and irrigated plantations only started in the 1980s [53]. A total of 37 small-scale projects were created covering ~10,000 ha of irrigated land and incorporating about 1200 small-scale growers into sugarcane production [53], which is the reason why there was no BC biomass emissions during the period of 1980. On the other hand, a high BC AOD value of ~0.014 (see Figure 2b) was observed in the year 2019 over the MP region. This BC emission was mostly from the sugarcane burning in the plantations. Higher BC AOD values (~0.012) in the KZN area were also observed during the 2019 period, particularly along the coast where sugarcane plantations are located. Calculating the difference between the years 2019 and 1980 shows an increase (+0.0002) in the BC AOD over the sugarcane plantation areas (see Figure 2c). The increase could be due to several factors, such as (1) increase in burning yield, and (2) favorable climate for burning. OC AOD distribution maps (see Figure 2–f) are similar to the BC AOD maps. The distribution of OC is observed over the plantation regions of the MP and KZN. Figure 2f demonstrates the increase of OC from 1980 to 2019. This is due to the increased activities of sugarcane burning over time. These results further show that the burning of sugarcane contributes immensely to the concentration of OC in those regions.

**Figure 3:**
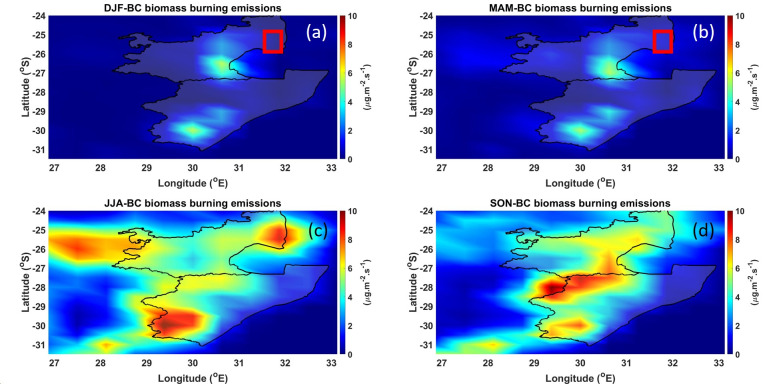
BC biomass emissions over the MP and KZN regions during the (**a**) DJF, (**b**) MAM, (**c**) JJA, and (**d**) SON seasons.

The averaged seasonal spatial distribution of BC biomass emissions over the MP and KZN provinces for the period of 1980 to 2019 is shown in Figure 3. The December–January–February (DJF) and the March–April–May (MAM) seasons show the lowest BC biomass emissions values of ~6 µg·m^−2^·s^−^^1^ (see Figure 3a,b). Moreover, the BC biomass emission values over the sugarcane plantations (red square) are >2 µg·m^−2^·s^−1^, indicating very minimal burning activities. Planting of sugarcane is usually from mid-February to early May; hence, there is very low emissions of BC during the two seasons. Moreover, December and January are the months with the highest precipitation. This is the period when the soil soaks up the rainwater, which is ideal for the preparation of the planting season. In the June–July–August (JJA) season (see Figure 3c), a plume of BC and a high values of BC biomass emissions (~9 µg·m^−2^·s^−1^) is observed in the MP region where the sugarcane plantations are situated. The JJA season is usually dry with lower temperatures. This is the season were most of the sugarcane burning is carried out. The September–October–November (SON) season (see Figure 3d) also experiences some sugarcane burning, but not as much as the JJA season; hence, the lower emissions (~6 µg·m^−2^·s^−1^) over the MP plantations.

**Figure 4:**
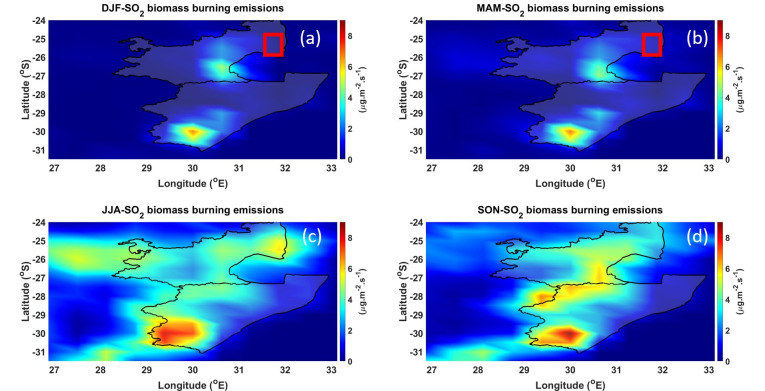
SO_2_ biomass emissions over the MP and KZN regions during the (**a**) DJF, (**b**) MAM, (**c**) JJA, and (**d**) SON seasons.

Similar to Figure 3, Figure 4 shows the averaged seasonal spatial distribution of SO_2_ biomass emissions over the MP and KZN provinces for the period of 1980 to 2019. The DJF and MAM seasons (see Figure 4a,b) show the least SO_2_ biomass emissions (>2 µg·m^−2^·s^−1^) in the MP plantations, and shows higher biomass emissions (~5.8 µg·m^−2^·s^−1^) in the JJA season (see Figure 4c). Moderate SO_2_ biomass emission (~4 µg·m^−2^·s^−1^) is observed in the SON season (see Figure 4d). During biomass burning, SO_2_ is produced by the flaming combustion process. Flaming combustion involves rapid reaction of O_2_ with gases evolved from the solid biomass fuel [51]. In this case, flaming combustion converts the sulfur in the fuel into highly oxidized gas, SO_2_. The contributions from flaming in the overall fire event are highly variable and depend on the fire intensity, fuel density, and fuel moisture, among other factors [13].

**Figure 5:**
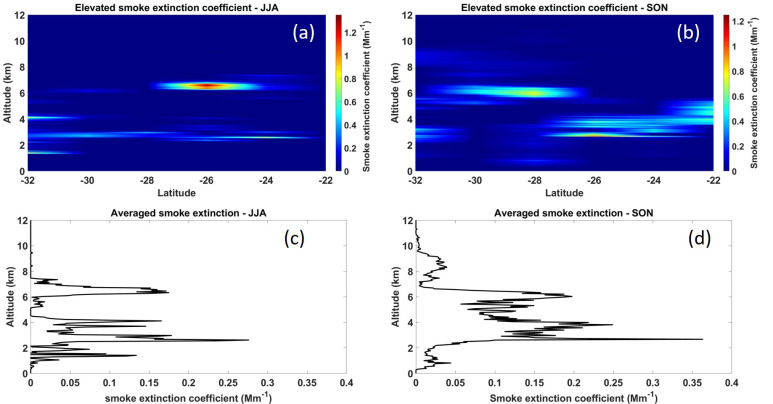
Averaged seasonal vertical height distribution of smoke aerosols over MP and KZN for the period of 2006–2019. Latitude-altitude cross sections of the smoke retrieved extinction coefficients in (**a**) JJA and (**b**) SON seasons. Altitude-resolved profiles of the mean smoke extinction coefficients in (**c**) JJA and (**d**) SON seasons.

The vertical distribution of aerosols is influenced by surface winds, turbulence, and aerosol size [54,55]. The more unstable the atmosphere, the more rapidly smoke is lifted and dispersed. Under stable conditions, smoke will not rise exceedingly, except from the heat of the fire and only for short distances. Figure 5 shows the averaged seasonal vertical height distribution of smoke aerosols over the MP and KZN regions. In the JJA season (see Figure 5a), a high extinction coefficient value of ~1.3 Mm^−1^ at a height of ~7 km is observed in the latitude between −27° and −25°. This region corresponds to the MP sugarcane plantations, and the smoke is due to the sugarcane burning, which is prominent in the JJA season. The mean vertical smoke extinction coefficient profile in Figure 5c shows that the highest smoke plume is detected at a height of ~7.3 km, while the highest smoke extinction coefficient is ~0.27 Mm^−1^ observed at a height of ~3 km. The smoke vertical profile further indicates that most of the smoke released from the burning resides around a height between 2.5 and 3.5 km. In the SON season (see Figure 5b), the highest smoke extinction coefficient value is ~0.8 Mm^−^^1^ at a height of ~6 km. On the other hand, a low smoke extinction coefficient value of ~0.1 Mm^−1^ is observed at a height between 8 and 10 km. The mean vertical smoke extinction coefficient profile in Figure 5d shows smoke plumes between 8 and 10 km, as well as dominant smoke plumes between 2 and 4 km, and between 5 and 6.5 km. Although the dominant season for sugarcane burning is in JJA, smoke emissions observed during SON are also a contribution from other agricultural burning activities.

### 4.2. Meteorological Conditions

**Figure 6:**
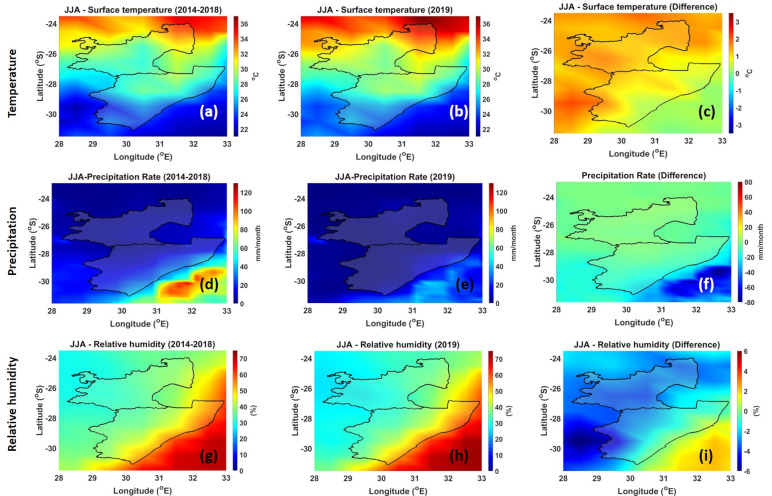
Spatial distribution of temperature (**a**–**c**), precipitation (**d**–**f**), and relative humidity (**g**–**i**) during the JJA season.

**Figure 7:**
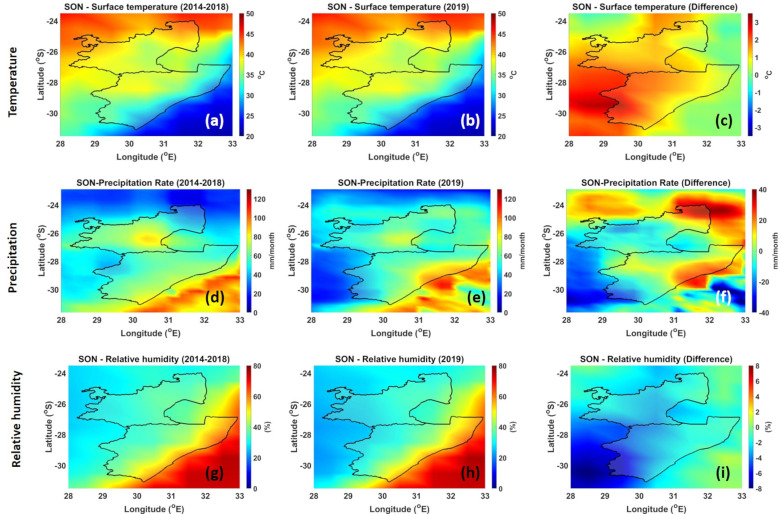
Spatial distribution of temperature (**a**–**c**), precipitation (**d**–**f**), and relative humidity (**g**–**i**) during the SON season.

Diurnal changes in temperature, relative humidity, wind speed, and direction influence fire behavior [56]. Temperature has a direct influence on fire behavior because of the heat requirements for ignition and continuing the combustion process. Precipitation has a direct and immediate effect on fuel moisture and relative humidity. Moist surfaces of fuels, due to relative humidity and precipitation, cannot ignite; thus, no burning can occur. Moreover, if the relative humidity is 100%, or close to it, the fuel will not dry and no burning can occur. Figure 6 and Figure 7 show the climatic conditions over the MP and KZN regions. The 5-year mean temperature (2014–2018) during the JJA season over the MP and KZN plantations is ~31 °C and 27 °C, respectively (see Figure 6a). During the 2019 JJA season (see Figure 6b), no drastic temperatures were observed; however, the differences (see Figure 6c) indicate a slight increase in temperature by +2 °C over the MP plantation region. The slight increase in the temperature can contribute to the drying of the immediate environment, making it favorable to fires. The low precipitation rate in the 5-year mean and the year 2019 (>1 mm/month) in the JJA season (see Figure 6d,e) also contribute to the dry conditions that are favorable for fires. Figure 6f, which illustrates the difference, shows no major changes in the precipitation rate especially over the sugarcane plantations areas. Relative humidity values over MP and KZN plantations for the 5-year mean and the year 2019 are ~40% and 60%, respectively (see Figure 6g,h). Relative humidity values between 20% and 60% can produce successful burns. Therefore, the observed relative humidity values also contribute positively for fires. Overall, the high temperature, low precipitation, and moderate relative humidity make it easy for the burning of sugarcane. These conditions serve well to start and continue the fire as desired by the farmers. Comparison between the SON and JJA season temperatures shows some variation. The 5-year mean and the year 2019 temperatures in the MP sugarcane plantation region are ~38 °C and ~30 °C in the KZN plantation region (see Figure 7a,b). Figure 7c indicates that there is a slight increase in temperature in the KZN plantation region, while there is no change in the MP plantation region. In the 5-year mean, during the SON season there is a significate change in the precipitation rate over the MP plantation (30 mm/month), and a precipitation rate of 80 mm/month is observed over the KZN region (see Figure 7d). During the 2019 period, precipitation rate for MP and KZN plantations were 50 mm/month and 80–110 mm/month, respectively (see Figure 7e). Figure 7f clearly shows an increase in the precipitation rate. The 5-year mean and the year 2019 relative humidity over the MP plantation was ~42% (see Figure 7g,h). There was also no major difference in the relative humidity (~55%) over the KZN plantations.

**Figure 8:**
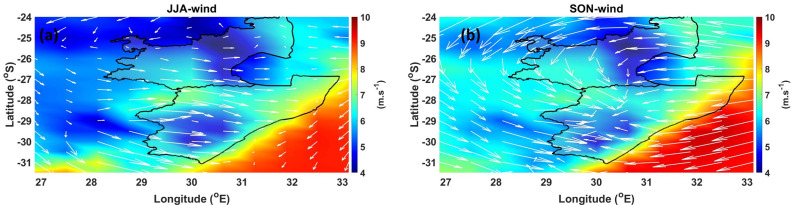
Average wind speed and direction (indicated as arrows) over the MP and KZN region during the (**a**) JJA, and (**b**) SON seasons.

Wind has a strong effect on fire behavior due to the fanning effect on the fire. It influences the rate of spread and intensity of the fire. Wind generally increases evaporation from damp surfaces by carrying away moist air and bringing in drier air. Furthermore, strong wind speeds can cause stalk breakage and lodging of sugarcane. Figure 8 shows the mean (1980–2019) seasonal wind speed and direction over the MP and KZN provinces during the JJA and SON seasons. During the JJA season (see Figure 8a), moderate wind speeds of ~6.5 m·s^−1^ in a north-easterly direction are observed over the MP plantations. The strong wind speeds indicate a stable atmosphere during this period, which is responsible for trapping pollution in this region [57]. Over the KZN plantations, moderate wind speeds of ~7 m·s^−1^ in a south-westerly direction is observed. Generally, in South Africa, strong winds and gusts during winter are usually caused by strong cold fronts, moving mostly over the southern half of South Africa, and also by the ridging of the high pressure systems behind the fronts [58]. The SON season (see Figure 8b) wind speeds patterns over the MP and KZN regions are similar to that of the JJA season. However, during the SON season, wind vectors indicting a strong high pressure like motion over the MP and KZN provinces are observed. This motion is likely responsible for the dispersion of pollutants (i.e., BC, SO_2_ and OC) in neighboring areas. Table 1 shows a summary of the climatic parameters over the MP and KZN provinces.

### 4.3. Trend Analysis and Dispersion of Pollutants

#### 4.3.1. Emissions from Sugarcane Burning

**Figure 9:**
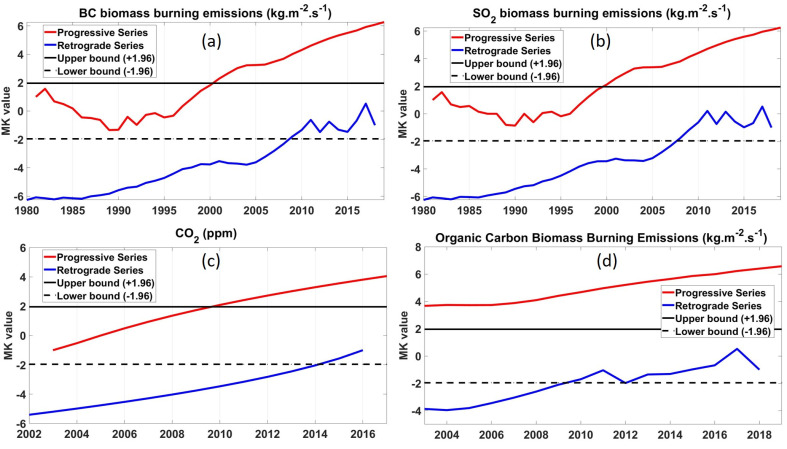
SQ–MK trends in (**a**) BC, (**b**) SO_2_, (**c**) CO_2_ and (**d**) OC over the MP and KZN regions.

A graphical illustration of the results of the SQ–MK test for BC, SO_2_, CO_2_, and OC are shown in Figure 9. Overall, the pollutants show an increasing trend. Figure 9a,b shows the trends of BC and SO_2_ emission for the period of 1980 to 2019, respectively. A significant increasing trend in BC and SO_2_ emission was observed from the year 1996. The increase in sugarcane production results in the increase in emission of BC and SO_2_ during harvesting. A decrease in BC and SO_2_ was observed from the years 1981 to 1990. The international anti-apartheid sanctions are responsible for the decline in the sugarcane industry [59]. The sanctions resulted in fewer tons of sugar being imported from South Africa [59]. This indirectly resulted in the decline in the production of sugarcane and, thus, less emission of BC and SO_2_ during the harvest period. The drought of 1983 also contributed negatively to the production of sugarcane [59], leading to less emissions of BC and SO_2_. One of the greenhouse gases (GHG) emitted from sugarcane burning is CO_2_.

Figure 9c shows an increasing trend of CO_2_ concentration from 2001 to 2017. There is increase in other activities, such as electricity production (e.g., burning coal, natural gases etc.) transportation, and industrialization that contribute to the increase in CO_2_ concentration. However, biomass burning also contributes to CO_2_ emissions. According to the Statistics South Africa report of 2017 [60], production of sugarcane fell by over half from 15.7 million tons to 7.5 million tons between 2007 and 2017. This implies that CO_2_ emissions from sugarcane burnings decreased over time. Therefore, other activities could have contributed more to the overall CO_2_ concentration observed in Figure 9c.

The major source of OC aerosols are biomass and fossil fuel burning activities. They are formed primarily by incomplete combustion or the oxidation of gas phase precursors [61], and they cool the atmosphere by scattering radiation [62]. The OC biomass burning emissions trend is shown in Figure 9d. A stable flat trend of OC emission for the period from 2003 to 2008 was observed, followed by a steady increase in OC biomass burning emissions for the period 2008 to 2019. Despite the reported negative production of sugarcane for the period 2007 to 2017 [60], sugarcane burning during harvesting still contributed significantly to the overall OC biomass burning emissions.

#### 4.3.2. Rainfall Trends

**Figure 10:**
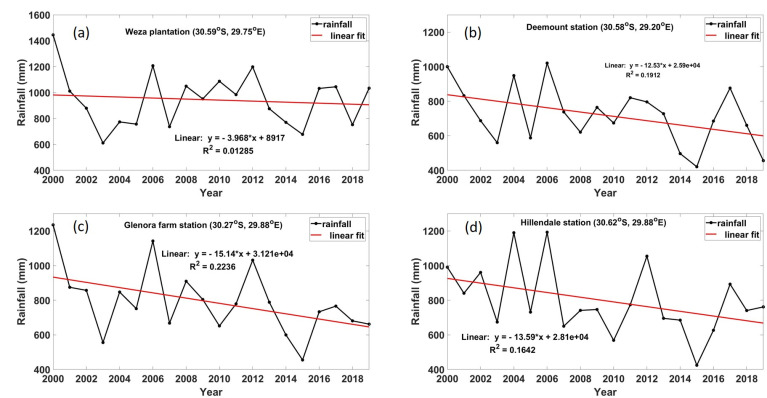
Linear rainfall trends in (**a**) Weza plantation, (**b**) Deemount station, (**c**) Glenora farm station, and (**d**) Hillendale station.

Sugarcane quality, in terms of sucrose, is affected by climatic factors, such as temperature, relative humidity, and rainfall [63,64]. Sugarcane is highly sensitive to water deficit, which can lead to a reduction of the sugarcane productivity by up to 60% [65]. On the other hand, excess water amounts can also have an increase in weeds, diseases, and insect pest on the sugarcane crop [66] and, thus, affect productivity. Therefore, adequate amounts of water are required for a healthy yield of the sugarcane crops. In KZN, 75% (9/12) of the plantations are rain-fed and 25% (3/12) use irrigation. Figure 10 shows a linear trend of rainfall at various meteorological stations close to the sugarcane plantations in KZN, for the period of 2000 to 2019. Over the Weza plantation meteorological station in Figure 10a, the linear fit shows a gradual decline of rainfall from ~1445 mm in the year 2000 to ~1034 mm in the year 2019 (~28.4% decline). The other meteorological stations, namely, Deemount, Glenora farm and Hillendale all show a sharp decrease in the rainfall in the 2000 to 2019 period. Deemount (see Figure 10b) shows a ~54.4% decline in rainfall, Glenora farm (see Figure 10c) shows a ~46.4 decline in rainfall, and Hillendale (see Figure 10d) shows a ~23.2% decline in rainfall. During the year 2000, a strong La Nina was experienced [67], which contributes to above to normal rainfalls. On the other hand, in 2019, a weak to mild El Nino was experienced [68], contributing to below to normal rainfalls. Therefore, the El Nino period is the main contributor to the overall decrease of rainfall in the study site. El Nino is also accompanied by dry conditions and warm to hot temperatures. This decreasing trend of rainfall might have a negative impact on sugarcane farming as it is heavily reliant on water.

### 4.4. Dispersion and Transport of Pollutants

**Figure 11:**
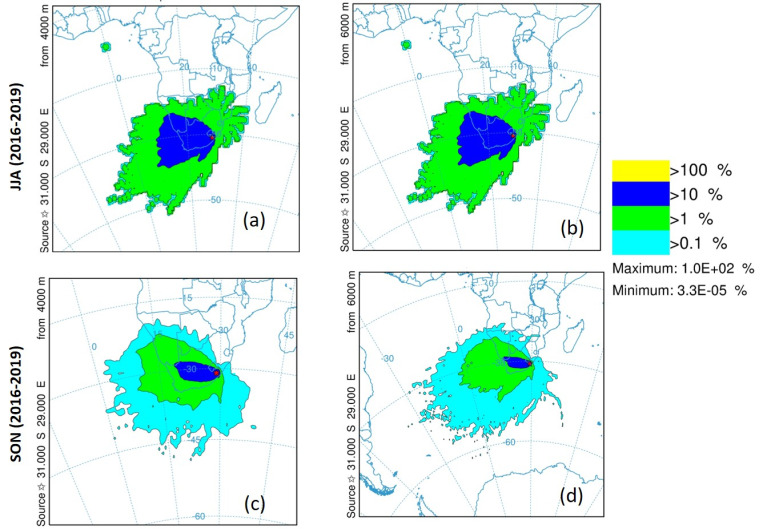
Seasonal trajectory frequency plots from a starting location of 31.000° S, 29.000° E during the period of 2016–2019 at (**a**) 4000 m and (**b**) 6000 m above ground level for the JJA season, and (**c**) 4000 m and (**d**) 6000 m above ground level during the SON season.

Air pollution distribution is affected by many factors, such as (1) meteorological conditions (i.e., wind speed, wind direction, and atmospheric stability); (2) the emission height (i.e., ground level sources, such as wildfires or high level sources, such as power station chimney stacks); and (3) geographical features. The movement of pollutants in the atmosphere is usually caused by deposition, transport, and dispersion. The deposition processes, including precipitations and scavenging, cause downward movement of pollutants in the atmosphere, which eventually remove the pollutants to the ground surface. The transport process is caused by the movement of a time-averaged wind flow. Lastly, dispersion results from local turbulence, that is, motions that last less than the time used to average the transport. Seasonal trajectory frequency plots from the southern KZN sugarcane plantations are shown in Figure 11. Two height levels (4000 and 6000 m) are chosen from Figure 5, which shows two strong smoke peaks at these heights. Figure 11a,c show air mass distributions at a 4000 m height during the JJA and SON seasons, respectively. During the JJA season (see Figure 11a), dominant air masses of frequency >10% travel towards the source in a south west direction over the Atlantic Ocean (but less than the 0° longitude line). Transboundary pollution is also observed with some air masses of frequency >10% traveling in a north–west direction over Namibia. The air masses of frequency >1%, traveling in a south west direction, traveled the longest distance close to 10° W longitude. These air masses transport smoke, BC, CO and other constituents from the burning sites of the sugarcane plantations. On the other hand, air masses of frequency >1% in the SON season (see Figure 11c) traveled shorter distances up to ~4° E longitude. The air masses of frequency >10% only reached the northern parts of South Africa. The air masses of a frequency of >0.1% which are not present in the JJA season, also traveled in a south west direction over the Atlantic Ocean up to ~0° longitude (~3000 km from the source). Air masses traveling over the neighboring countries, i.e., Namibia and Botswana are also observed. This confirms the transboundary pollution from South Africa during this period. During the SON season, air masses of frequency >0.1% at 6000 m (see Figure 11d) shows that pollutants from the sugarcane biomass burning sites traveled the furthest up to ~30° W longitude (~6660 km from the source). The air masses of frequency >0.1% and 10% are similar to that of Figure 11c. The air masses patterns at 6000 m in the JJA season (see Figure 11b) are similar to those in Figure 11a.

## 5. Conclusions

The South African sugar industry makes an important contribution to the South African economy. The sugar industry creates employment in rural and deep rural areas where there are often little or no economic activities. However, the process of sugarcane burning for harvesting purposes poses a health risk to the surrounding communities. This study shows an increase over time (from 1980–2019) in emissions from sugarcane burnings. Specifically, increases in the BC, CO_2_, and SO_2_ species are observed. The WHO regional office for Europe shows that long-term average BC exposure can lead to cardiopulmonary mortality [69], whereas increases in CO_2_ can lead to regional climate change [70]. The increase in emissions between 1980 and 2019 was caused by the emergence of several small sugarcane farmers. These collectively have led to an increase in biomass burning. In the MP province smoke plumes are observed at heights of 4 and 6 km, while in the KZN province, smoke plumes are observed at heights of 4, 6, and 9 km. Atmospheric stability and wind speeds are the major influencers of the vertical distribution of the smoke. Overall, the atmospheric stability plays a critical role in the vertical and spatial distribution of the atmospheric constituents from the biomass burning. During the burning of sugarcane plantations for harvesting, meteorological conditions play a vital role in the burning process. During the JJA season in KZN, moderate temperatures of ~25 °C, precipitation of ~0 mm/month and moderate relative humidity of ~60%, make the conditions ideal for burning. Moderate wind speeds of ~7.5 m·s^−1^ also aid in the fueling of the fire. The decreasing rainfall trend in the KZN makes it even drier during the JJA season, favoring the burning. The constituents from the biomass burning are transport to neighboring countries and over the Atlantic Ocean as far as ~6600 km. Because of the transport of these pollutants, surrounding areas and far areas are impacted by these pollutants. This might lead to health problems and climate variability issues.

## Figures and Tables

**Table 1:** Summary of the averaged climatic parameters over the Mpumalanga and KwaZulu Natal provinces.

	Mpumalanga Province		KwaZulu Natal Province	
Parameter	JJA Season	SON Season	JJA Season	SON Season
Temperature (°C)	31	38	25	28
Precipitation (mm/month)	0	50	0	80
Relative humidity (%)	50	52	60	52
Wind speed (m·s^−1^) and direction	6.5 (north-easterly)	6.5 (westerly)	7–8 (south-westerly)	7–8 (westerly)

## Data Availability

BC, SO_2_, OC, temperature, precipitation, relative humidity, and wind were obtained from this link: https://giovanni.gsfc.nasa.gov/giovanni/ (last accessed 17 May 2021). CALIPSO smoke aerosol coefficient data were downloaded from this link: https://urs.earthdata.nasa.gov/ (last accessed 24 May 2021).
